# Personalized medicine in Europe: not yet personal enough?

**DOI:** 10.1186/s12913-017-2205-4

**Published:** 2017-04-19

**Authors:** Antonello Di Paolo, François Sarkozy, Bettina Ryll, Uwe Siebert

**Affiliations:** 1 Department of Clinical and Experimental Medicine, Section of Pharmacology, University of Pisa, Via Roma 55, 56126 Pisa, Italy; 2 FSNB Health & Care, Carenity, Paris, France; 3 Melanoma Patient Network Europe; Evolutionary Biology Centre, Uppsala University, Uppsala, Sweden; 4 Department of Public Health, Health Services Research and Health Technology Assessment, UMIT – University for Health Sciences, Medical Informatics and Technology, Hall in Tyrol, Austria; 5 Area of Health Technology Assessment, ONCOTYROL – Center for Personalized Cancer Medicine, Innsbruck, Austria

**Keywords:** Personalized medicine, Definition, Patient stratification, Patient preferences

## Abstract

**Background:**

Personalized medicine has the potential to allow patients to receive drugs specific to their individual disease, and to increase the efficiency of the healthcare system. There is currently no comprehensive overview of personalized medicine, and this research aims to provide an overview of the concept and definition of personalized medicine in nine European countries.

**Methods:**

A targeted literature review of selected health databases and grey literature was conducted to collate information regarding the definition, process, use, funding, impact and challenges associated with personalized medicine. In-depth qualitative interviews were carried out with experts with health technology assessment, clinical provisioning, payer, academic, economic and industry experience, and with patient organizations.

**Results:**

We identified a wide range of definitions of personalized medicine, with most studies referring to the use of diagnostics and individual biological information such as genetics and biomarkers. Few studies mentioned patients’ needs, beliefs, behaviour, values, wishes, utilities, environment and circumstances, and there was little evidence in the literature for formal incorporation of patient preferences into the evaluation of new medicines. Most interviewees described approaches to stratification and segmentation of patients based on genetic markers or diagnostics, and few mentioned health-related quality of life.

**Conclusions:**

The published literature on personalized medicine is predominantly focused on patient stratification according to individual biological information. Although these approaches are important, incorporation of environmental factors and patients’ preferences in decision making is also needed. In future, personalized medicine should move from treating diseases to managing patients, taking into account all individual factors.

**Electronic supplementary material:**

The online version of this article (doi:10.1186/s12913-017-2205-4) contains supplementary material, which is available to authorized users.

## Background

With many new drugs designed against specific targets the use of biomarkers will increasingly allow patients to receive the drug most likely to be effective for their disease. In parallel, there is an increasing awareness that the different administration regimens and side effect profiles associated with particular therapies can impact health-related quality of life (HRQoL) in ways that vary among individuals. Differences also exist among patients with regard to benefit–harm trade-offs and willingness to obtain the best possible care, and individuals may benefit from holistic solutions adapted to their individual profile and preferences. These approaches could be applied to the majority of procedures and pharmacological agents, not just targeted therapies. Using biomarkers to target therapies is regarded by some as a way towards a more efficient and cost-effective healthcare system [1–4]. This is particularly important as across Europe ageing populations and the increasing prevalence of chronic disorders are placing healthcare budgets under pressure. As a result, healthcare systems are increasingly mindful of budget impact and cost-effective usage of expensive innovative agents.

Personalized medicine (PM) is a relatively young field, although there have been a substantial number of recent publications in the area of PM and how it could be facilitated and implemented [3, 5, 6]. Different stakeholders’ views on PM may reflect different aspects of the overall concept, and a comprehensive overview of what is understood by the term PM (also referred to as ‘stratified medicine’), and exactly what benefits and challenges may be associated with its implementation, is needed.

Therefore, the purpose of this research was to provide an overview of the concept of PM in Europe, with a particular focus on how patient segmentation is currently performed in nine countries with different healthcare systems: Austria, France, Germany, Hungary, Italy, the Netherlands, Spain, Sweden and the UK. In addition, we sought to identify some of the challenges and opportunities of PM in terms of providing the greatest possible value both for individual patients and society.

To meet these goals, we first conducted a targeted literature review of selected health databases and grey literature with the aim of collating qualitative information regarding the definition, process, use, funding, impact on health technology assessment (HTA) evaluation and challenges associated with PM. We decided to perform a qualitative literature review limiting the search to the selected European countries, and the findings were taken into account during the elaboration and validation of interviews. Second, because PM is an evolving field, and to capture an up-to-date picture of how PM is viewed across Europe, we carried out in-depth qualitative interviews with a range of experts with HTA, clinical provisioning, payer, academic, economic and industry experience, while members of patient associations were also interviewed. Finally, we describe some of the challenges that will need to be overcome in order to integrate patients’ preferences into PM decision-making processes.

## Methods

### Literature review

Searches of the MEDLINE and Embase databases were carried out between October 2014 and February 2015 using terms specific to PM, including ‘personalized medicine’, ‘individualized medicine’, ‘stratified medicine’, ‘segmented medicine’ and ‘targeted therapies’ according to the PRISMA guidelines (see Additional file 1 for full search terms). Electronic database searches were restricted to English language only; dates searched were 2010 to present but limiting the survey to the nine selected European countries. Similarly, grey literature searches were performed for the nine countries of interest – these searches were not limited to the English language and relevant materials were translated where possible.

Literature was selected for inclusion in the review if it addressed topics such as the definition of PM; methods for patient segmentation; current HTA, reimbursement, pricing and funding processes for PM; and the impact of these on patient access (see Additional file 1 for complete list).

The literature search in MEDLINE generated 1009 potentially relevant titles and/or abstracts and was conducted in October 2014. The Embase search generated 8580 potentially relevant titles and/or abstracts and was conducted in February 2015. Due to the large number of results in the Embase search the number of publications from this specific search was reduced by first filtering the initial results based on duplicates and additional search terms (see Additional file 1).

Both the results from the MEDLINE search and the remaining titles and/or abstracts from the Embase search were filtered based on their relevance to the research questions. For all relevant titles and abstracts, full papers were reviewed, and relevant data were extracted for evaluation. In case of doubt regarding the relevance of a title abstract or paper, a second researcher was consulted.

Based on those papers from the MEDLINE search that were most interesting or provided information and/or data that were scarce within the literature results, a citation search was conducted. In addition to the literature searches carried out in MEDLINE and Embase, the initial search terms were also used to search for literature on relevant websites. The identified literature was screened, reviewed and filtered by a second researcher, who also extracted data from the literature deemed relevant.

Finally, the retrieved literature was analysed to identify possible general themes related to PM, such as those concerning the use of (bio)markers or patients’ preferences, which could refer to “traditional” or innovative attitudes in PM, respectively.

### Expert interviews

Semi-structured, anonymized interviews were conducted with experts from nine European countries, as well as three experts from European patient organizations, and one representative of the European Federation of Pharmaceutical industries and Associations (EFPIA). Experts were recruited from a BresMed internal contact list, and additional participants were recruited via snowball sampling. Interview recruitment was double blinded: the experts were not aware of the identity of the sponsor or of the Authors, and *vice versa*, and this procedure was explained to the experts during the administration of the informed consent. Of note, the privacy of experts was protected by excluding any information that could identify the interviewees while the complete anonymization of collected data was adopted before every analyses were carried out and findings discussed.

An interview guide (see Additional file 2) was developed with open and closed questions based on themes identified from the published evidence base and any obvious gap in the collected and reviewed literature. Pilot interviews were conducted in February 2015 to address any unclear or duplicated questions, and interviews with experts were conducted between April and June 2015. Interviewees were briefed about the objectives of the research and gave their informed verbal consent to participate and for the interviews to be recorded. Interviewees were asked about aspects of PM including definitions, patient segmentation, assessment and implementation of PM, the challenges they perceived personalized medicine to be facing, potential solutions, and expectations of the future of PM. The interviewer asked general questions without forcing the expert toward a pre-classified response.

Qualitative analysis was carried out through coding the transcripts using a software program (NVivo 10, QSR International) to identify any trends, differences and similarities specific to the HTA and reimbursement processes, decision making, challenges, and promoting market access across the study countries. For presentation of the results, anonymity of participants was protected by removing names and creating broad interview categories (e.g., clinical expert or economic expert).

## Results

### Literature review

The literature review revealed that there is no overall consensus on how to define PM, with a number of different definitions in use [1–3, 5, 7–41] – in the literature these ranged from “*targeted treatment tailored to the genetic makeup of individual tumours”* [27] to “*the idea that medicines and other health technologies including the prediction of individual risk may be customised to each person’s specific genetic, physiological or psychological characteristics*“[41]. In particular, the retrieved literature referred to two different semantic approaches for PM: patients’ stratification (18 out of 38 articles), that is grouping individual patients in subpopulation according to their probability to have a therapeutic benefit from a drug or regimen, and treatment tailoring (19 out of 38), that is, the individual status of a patient (i.e., disease characteristics or subject’s genotype/phenotype) is the rationale basis for drug choice. Interestingly, two papers did identify PM as a procedure that necessitates the development of targeted agents [10, 18].

Most studies (30 out of 38) described the use of diagnostics and individual biological information, including clinical characteristics, genetic disposition and biomarkers, in order to target therapies to the patient and disease, with the aim of improving outcomes and reducing side effects (definitions are presented in Additional file 3 as exact quotations from the original articles).

Reviewing the definitions, it clearly appears that both stratification and tailoring may be based on tests. Indeed, much of the published literature on physiology-based PM concerns the use of genetic information and biomarkers to select patients for whom a particular treatment is appropriate. Effective use of biomarkers requires the availability of validated diagnostic tests, and a number of drug treatments have specific companion diagnostics (CDs) [28, 42–44]. Ensuring that CDs are accessible is a major challenge in the use of physiology-based PM, and the lack of simultaneous availability of both a drug and its CD is a frequent issue in clinical practice. Processes for assessing the clinical value and cost-effectiveness of CDs are not well established, and a major limitation is that assessments of drugs and CDs are often conducted separately, and fail to capture the full benefits of these products [28, 45]. The use of CDs may also be associated with ethical issues (Table 1) [46].

**Table 1:** Ethical issues associated with the use of companion diagnostics [46]

Issue	Concern
Informed consent	• The process of getting consent from the patient for testing is both lengthy and complex
Data management	• Testing generates data which should be identifiable and integrated into datasets of genomic and health information • Interpreting test data requires skilled professionals who are able to interpret and translate the data to patients
Communication of results	• Translating the results to patients is becoming increasingly difficult, as the number of biomarkers being tested by a single test is constantly increasing • Testing can provide incidental findings and variants of unknown significance, knowledge of which can affect a patient’s well-being • Patients have concerns about privacy and the possible disclosure of genetic information. They have concerns about who sees their results during the analysis process and a potential risk of discrimination if such information is known
Cost and equity issues	• The costs for targeted therapies are usually high; drugs and accompanying tests might not always be covered by health insurance, which can limit patients’ access to treatment • High costs increase the imbalance in access to new and better treatments as the identification of new biomarkers and treatments continues
Guidelines	• There is a lack of guidelines regarding implementation of testing

It is worth noting that other stratification criteria used are mostly demographic, and include factors such as age and sex. A recent study by Schleidgen et al. (2013) [3] has suggested a definition of PM as follows: “*personalized medicine seeks to improve stratification and timing of health care by utilizing biological information and biomarkers on the level of molecular disease pathways, genetics, proteomics as well as metabolomics*.”

By contrast, Rogowski et al. (2015) [38] reported the results of a series of structured workshops, organized by the International ONCOTYROL Expert Task Force [47], which supported the division of PM into physiology- and preference-based PM [38]. The first category, also termed ‘stratified medicine,’ refers to segmentation of patients according to their genetic makeup, while the second refers to the tailoring of treatment according to patients’ preferences.

In contrast to physiology-based approaches, a few papers mentioned terms such as patients’ individual needs, beliefs, behaviour, values, wishes, utilities, environment and circumstances in their definitions of PM [5, 8, 15, 16, 26, 38, 41]. Damato et al. (2013) [16] defined PM as “*the tailoring of therapy to the needs, wishes, fears, and condition of the patient, also taking account of the individual’s circumstances*.” The description by Rogowski et al. of preference-based PM as developed by the ONCOTYROL Expert Task Force included both revealed preferences such as adherence to treatment and stated preferences such as attitude to risk [38].

In addition, a study by the European Commission recommended that the use of patient preferences in PM should be increased [19]. Making treatment decisions in conjunction with patients’ views does occur to a greater or lesser extent in particular settings; however, there was little evidence in the literature for formal incorporation of patient preferences into the evaluation of new medicines.

Finally, the survey showed that PM has mainly a predictive role (29/38 articles), being useful to anticipate the efficacy of treatments, whereas only two of them [18, 31] included the possibility to prevent “side effects” within the definition. At the same time, only 13 papers stated that PM has also a preventive role because it may determine the individual “susceptibility to a particular disease”. In turn, that knowledge allows the adoption of “preventive interventions” aimed at reducing the risk.

### Expert interviews

In total, 34 interviews were conducted with three patient association members, one member of EFPIA, seven academics, five clinicians, nine economists, five payers and four providers (Table 2). The qualitative analysis of interviews did not result in the identification of clear trends across countries, and this was likely dependent on the limited number of experts. However, none of the interviewees gave a specific definition of PM, even if most responses described approaches to stratification and segmentation of patients based on genetic markers or CDs in agreement with the findings of literature survey. All respondents mentioned the use of tests or CDs to identify sub-groups, to stratify patients to the right treatments, or to identify patients who might benefit the most from treatments. The majority of experts (13 of 17 [76%]) believed that CDs were important, but respondents generally felt valuing these tools to be challenging, as their value is intrinsically linked with the corresponding pharmaceutical product.

**Table 2:** Distribution of external experts

Country	Academic	Clinical	Economic	Payer	Provider	Patient rep.	EFPIA
Austria	1	1	1			3	1
France	1	1	1	1	1		
Germany	1	1	1	1	1		
Hungary	1		1				
Italy	1		1				
Spain			1	1			
Sweden			1				
The Netherlands	1	1	1	1	1		
UK	1	1	1	1	1		

EFPIA, the European Federation of Pharmaceutical Industries and Associations; rep., representative

Patient representatives were from European groups

When asked about motivations for the segmentation of patients (Table 3), the majority of experts used terms associated with improvement of outcomes (14 of 20 [70%]) or optimization of side effect profiles (11 [55%]). Another common reason for segmenting patients was to avoid overtreatment or wasting resources (10 [50%]). In addition, one of the patient representatives mentioned that PM can also empower patients and clinicians: *“… by giving them knowledge about their condition, about their genes, about the options they have and making them more powerful when they are making informed choices.”* Another patient representative expressed concern that patient segmentation should be conducted carefully: *“… I think there is a risk that precision medicine will be seen to be an excuse for rationing, rather than a clinical tool for better patient care.”* Only two experts mentioned HRQoL.

**Table 3:** Interview respondents’ views on motivation for patient segmentation

Benefits	Experts
Avoiding side effects/optimize side effect profile	Academics (3), clinical experts (2), economic experts (3), EFPIA representative (1), patient representatives (2)
Avoiding waste of resources/over-treating/selecting only patients who need it	Academic (1), clinical experts (2), economic experts (3), EFPIA representative (1), patient representatives (2), payer (1)
Improved outcomes in terms of effectiveness/efficacy	Academic (1), clinical expert (1), economic experts (3), patient representative (1), payer (1)
Better outcome/benefit/response rate (not specified)	Academic (1), clinical experts (2), economic experts (2), provider (1)
Improved cost-effectiveness/value for money	Clinical expert (1), economic experts (3), payer (1)
Reduce costs	Economic experts (3), payer (1)
Improved length of life	Academic (1), clinical expert (1), economic expert (1)
Improved quality of life	Academic (1), economic expert (1)
Free-up time from clinicians	Patient representative (1), payer (1)

EFPIA, the European Federation of Pharmaceutical Industries and Associations

In total, eight respondents (of 34 [24%]; two clinicians, two economists, two payers, one provider, and one academic) mentioned the incorporation of factors such as environment and social setting in the treatment process, but only one (payer [3%]) specifically mentioned patient’s preferences. By contrast, two experts (6%) stated that they did not believe patient preferences to be important for patient segmentation.

With regard to challenges in PM, several respondents highlighted ethical concerns about patient segmentation. For example, one clinician felt that using factors such as age to stratify patients could be considered discriminatory. Others noted that physicians can not force patients to undertake tests; if they refuse to take tests, they cannot necessarily be refused treatment. There was a clear feeling that more needs to be done to find reliable ways to segment patients (7 of 15 [47%]), both to identify higher-risk patients and to prevent potentially inappropriate restrictions on access.

One limitation of PM that was mentioned is the potential for increased uncertainty when estimates of treatment effects are based on small patient populations – for example, “*The trouble is that once you get into personalized medicine, each time you look at a smaller subgroup of an extra factor in terms of the evidence of benefit then you get more uncertainty*” (clinician). Furthermore, the implementation of stratification using CDs could contribute to uncertainty (academic, clinician), and increasing medicine complexity could lead to errors (clinician). It was noted that use of real-world evidence to supplement gaps in randomized clinical trials could generate significantly more data and find better biomarkers that can help guide treatment. A number of experts also noted a need for the collection of more information on biomarkers and disease (two payers), more genomic sequencing (clinician), as well as more and larger trials (payer, clinician).

## Discussion

Current definitions of PM focus either on demographic criteria or on the use of clinical and biological information, including genetic disposition and biomarkers, to improve stratification of patients to receive optimized treatments. The results of the literature review and expert interviews described here show that these approaches – ‘physiology-based PM’ – are receiving a significant level of attention in the literature and in clinical practice. Although the findings of our survey were analysed in a qualitative way, they matched those obtained by Schleidgen and colleagues [3], who analysed the literature, categorized and quantified the results until they concluded that PM “seeks to improve stratification and timing of health care” by using biological information and biomarkers [3]. The use of biomarkers in treatment decisions is important, and there is a clear need to improve the systems for making relevant tests for biomarkers or specific genetic characteristics available alongside new medicines. In particular, the assessment of the value of a medical treatment and its associated CD together rather than separately is likely to simplify the assessment of both.

It is worth noting that part of our findings suggest that PM could be a holistic methodology, which is “centered around the needs of individual patient” as stated before [3]. Indeed, although the holistic vision of PM is confined to a reduced number of published articles [13, 38], it is plausible that offering more therapeutic options, as it occurred in some diseases over the last few years, means giving a growing weight to patients’ needs during the decision-making process.

What has been recorded during interviews largely matches the findings of literature survey, as briefly presented in the paragraph above. However, some important themes have been identified in the interviews. First, the majority of experts did agree with the physiological definition of PM, which should be based on a test (whatever the assay could be) to improve patients’ stratification and treatment, both in terms of efficacy and tolerability. This definition matches that synthesized by Schleidgen et al., (2013) [3], which excludes the possibility that patients’ preferences could have an influence on therapeutic management. This is not surprising, and it could depend on several different factors, such as the need to reduce uncertainty and to objectively stratify patients according to their individual probability or risk of experiencing a therapeutic benefit or toxic effects, respectively. Interestingly, further points of discussion about PM were identified by the interviewees. Indeed, some experts referred to the application of PM protocols as possible causes of uncertainty, while a patients’ representative stated that PM could impede some patients to get access to potentially beneficial treatments. Those issues are very similar and they are not completely new for medicine, because the adoption of some screening procedures could result in erroneous values, thus denying therapeutic opportunities to some patients. It is likely that the development of a CD in parallel with the drug may improve the technical characteristics of the test.

The panorama is completed by the impact of PM for patients, the third aspect. Indeed, disclosing and discussing results of tests with the patients will turn into an informed decision making, as one patients’ representative stated. It is interesting to note that although not explicit, every shared decision making is based on the doctor-patient relationship and does include patient’s preferences [48]. In a wider view, the discussion concerning a pharmacological option for a disease does mean taking patient’s preferences into account. Indeed, Schleidgen and colleagues discussed about the possibility that PM “is not a new concept as medicine has always been individualized” [3]. Unfortunately, that assumption does not match with the influence of individual needs and beliefs on treatments that was neglected by the majority of experts.

Overall, the present study suggests that there was little mention in the literature or in the interview results of the use of patients’ behaviour, beliefs, values, personal environment and individual preferences in treatment decisions and the related trade-offs. In particular, there was little evidence for formal incorporation of patient preferences, utilities and social/cultural characteristics into the evaluation of new medicines. Few of the interviewees mentioned HRQoL, suggesting that most stratification is based on individual, but not personal, factors.

We believe that to become truly personalized, medicine will need decision making processes that can help define therapeutic solutions adapted to an individual patient’s profile, including not only their clinical characteristics or genetic disposition, but also their environment and individual preferences.

In addition to disease characteristics and the results of diagnostic tests, it will be important to take into account other patient’s characteristics. Medication adherence and disease management can be affected by patients’ personal environment, and by behavioural factors, such as their knowledge, abilities, occupation, social status, cultural background and beliefs [49]. Patients may also have individual preferences for particular treatment modalities, the avoidance of certain side effects [50], and the benefit–harm trade-off of interventions, and may differ in the level of priority they give to health compared with other problems.

PM will therefore need to actively involve patients in the choice of the optimal therapeutic solution for them – to achieve this patients will need information about the disease, the effectiveness of each therapy and the corresponding side effect profiles in order to make an informed decision. In this way, the goal of healthcare providers and patients will be to minimize side effects, secure outcomes and benefits, improve efficacy and HRQoL, and optimize the benefit–harm balance for each individual. By matching the characteristics of the selected therapy to a patient’s lifestyle and preferences, common reasons for non-adherence to prescribed treatments may be avoided, potentially increasing the expected therapeutic benefits, as well as optimizing the use of available healthcare resources. In principle, PM should therefore benefit both the individual patient and society. From a healthcare system perspective, the benefits of PM are likely to be improved outcomes and increased cost-effectiveness through selection of the best treatment for each patient.

Personalization of medicine may therefore take a number of forms not including only a laboratory test (i.e., a biomarker) but also individual preferences. For example, androgen-deprivation therapy is an option for the treatment of localized prostate cancer, but can cause impairment of urogenital function [51] as well as radical prostatectomy [52, 53]. The benefit–harm ratio for aggressive treatments may therefore be very different between individual patients, depending on their lifestyles and individual preferences – this could lead to very different estimates of patient-relevant outcomes and cost-effectiveness, depending on what patient population is being considered [54]. In a wider view, the application of PM may also encompass, for example, not only patients’ preferences but also social constraints [55], risk perception of a disease and, in an informed way, decision making [56].

A major challenge in the implementation of PM based on patients’ preferences as well as their physiology will be how best to capture preferences in the assessment of new pharmaceutical products and diagnostics. Patients’ lifestyle, occupation and personal preferences may significantly change the value of a particular intervention from individual to individual and for the same individual on the basis of different situations/conditions, thus requiring a systematic classification (taxonomy) of outcomes or quality of life according to patients’ needs. These differences are likely to add to the complexity of HTA processes, particularly in systems that rely on willingness-to-pay thresholds in terms of cost per quality-adjusted life-year to guide decision making. Once drugs are available for use based on both the results of diagnostic tests and patients’ wishes, there will be a need for appropriate information on treatment effectiveness and side effect profiles that can be provided to patients, and for validated tools that healthcare providers can use to collect individual patients’ preferences and their explicit trade-offs in an unbiased manner. However, that approach will likely depend on the availability of large databases on which tools could be elaborated and validated. Demonstrating the benefits of personalization of medicine beyond the use of biomarkers will necessitate substantial evidence generation after a product’s launch, through both pragmatic trials and observational real-world studies. Finally, effective personalization of medicine based on patients’ environment and preferences will require an understanding of how health outcomes are affected by patients’ behaviour at individual and collective levels. Therefore, it is likely that the implementation of such an approach in the HTA processes will require several substantial changes.

The present study has several limitations. The literature search was not exhaustive, because the search terms were not designed to identify publications in areas such as behavioural science. Behavioural concepts such as patient activation may have an important role to play in the development of PM [57]. In addition, while attempts were made to interview experts with a range of backgrounds, the number of interviewees was limited. In particular, only two experts from Hungary, Italy and Spain, and one from Sweden, were interviewed. Interview responses may therefore not be fully representative of decision makers in all countries. Similarly, the number of patient group representatives was small, and their responses may not sufficiently reflect patients’ perspectives. Finally, all interviewees did not answer some of the questions, although all experts did respond to items in each section of the interview guide.

## Conclusions

In conclusion, the results of a literature review and a series of expert interviews have shown that PM is focused on patient stratification according to individual biological and clinical information. Although these approaches are important, incorporation of environmental factors and patients’ preferences in decision making is also needed. We believe that, rather than reducing patients to their disease, they must be viewed holistically as human beings with individual values within an overall social context. In future, PM should move from treating diseases to managing patients, taking into account all individual factors.

## Additional files

Additional file 1: Summary of literature review methods and PRISMA flow diagram. (PDF 138 kb)

Additional file 2: Interview questionnaire. (PDF 130 kb)

Additional file 3: Definitions taken from the literature. (PDF 71 kb)

## Abbreviations

CD: Companion diagnostic
EFPIA: European federation of pharmaceutical industries and associations
HRQoL: Health-related quality of life
HTA: Health technology assessment
PM: Personalized medicine
