# Supplementary material for: Personalized medicine in Europe: not yet personal enough?
Source: BMC Health Serv Res. 2017 Apr 19;17:289. doi: 10.1186/s12913-017-2205-4 (PMC5395930; doi:10.1186/s12913-017-2205-4)

Additional file 1 – summary of literature search methods

Selected countries	Austria<br><br>France<br><br>Germany<br><br>Hungary<br><br>Italy<br><br>Spain<br><br>Sweden<br><br>UK
Limits	General literature search: 2010 – Current
Publication types	Case studies<br><br>Published literature reviews<br><br>Published guidelines/recommendations<br><br>Published reports<br><br>Interview transcripts<br><br>White papers<br><br>Research articles
Global electronic databases	Embase, MEDLINE
General websites	ISPOR/ ASCO/ ESMO/ SMDM/ iHEA<br><br>WHO<br><br>HTAi<br><br>OECD

	EFPIA<br><br>DG SANCO<br><br>European Commission<br><br>ONCOTYROL<br><br>CANCERGEN<br><br>PHARMGKB<br><br>EMA<br><br>Patient and Clinician Engagement group (PACE)<br><br>European Hospital Association
Country specific websites	NICE/ NHS/ NATIONAL CANCER FUND (UK)<br><br>HAS/ CEPS/ ANSM/ INCa (France)<br><br>IQWiG/ DIMDI/ G-BA (Germany)<br><br>AIFA (Italy; plus regional websites)<br><br>AETS (Spain; plus regional websites)<br><br>SBU (Sweden)<br><br>LBI-HTA (Austria)<br><br>Austrian Social insurance (www.sozialversicherung.at)<br><br>NHIF (Hungary)<br><br>SMC (Scotland)<br><br>ZINL (Netherlands)
Search Terms	Personalized Medicine/Care<br><br>Individualised Medicine<br><br>Stratified Medicine

	Segmented Medicine<br><br>Targeted Therapies<br><br><br>Any of the above with:<br><br>+ definition<br><br>+ individualised/stratified/specialised/segmented/integrated<br><br>+ genomics/proteomics/metabolomics<br><br>+ behavioural/psycho-social/social/individual needs<br><br>+ oncology/cancer/tumours/neoplasm<br><br>+ technology/diagnostics<br><br>+ health care/pharmaceuticals<br><br>+ Europe/France/Italy (including regions)/Germany/Spain (including regions)/ UK/England/ Austria/ Sweden/ Hungary<br><br>+ expectations<br><br>+stakeholders/payers/providers/clinical/patients/public/consumer /patient satisfaction<br><br>+ pharmaceutical + medical devices/products/devices/treatment/novel/innovative/companion diagnostics<br><br>+ outcomes<br><br>+ ethics/ethical<br><br>+ Challenges<br><br>+ Patient's Preferences

	+ Economic Models<br><br>+ Health Technology Assessment/HTA/Funding/Pricing/Reimbursement<br><br>Companion Diagnostics<br><br>Challenges
Criteria for inclusion in the review – topics discussed	Definition of personalized medicine<br><br>Predominant components of personalized medicine<br><br>Methods for patient segmentation, and current best practices<br><br>Current HTA, reimbursement, pricing and funding processes for personalized medicine, and impact of these on patient access<br><br>Specific tools or methods used to inform investment decisions surrounding personalized medicine<br><br>Challenges to the implementation of personalized medicine

AETS, Agencia de Evaluación de Tecnologías Sanitarias; AIFA, Agenzia Italiana del Farmaco; ANSM, Agence nationale de sécurité du médicament et des produis de santé; ASCO, American Society of Clinical Oncology; CANCERGEN, Cancer Genetics Network; CEPS, Comité Économique des Produits de Santé; DG SANCO, European Commission Directorate General for Health and Consumer Protection; DIMDI, German Institute of Medical Documentation and Information; EFPIA, European Federation of Pharmaceutical Industries and Associations; EMA, European Medicines Association; ESMO, European Society for Medical Oncology; G-BA, Gemeinsamer BundesAusschuss; HAS, Haute Autorité de santé; HTA, Health Technology Assessment; HTAi, Health Technology Assessment international; iHEA, International Health Economics Association; INCA, French National Cancer Institute; IQWiG, Institüt für Qualität und Wirtschaftlichkeit im Gesundheitswesen; ISPOR, International Society for

PharmacoEconomic and Outcomes Research; LBI-HTA, Ludwig Boltzmann Institute for Health Technology Assessment; NHIF, National Health Insurance Fund; NHS, National Health Service (UK); NICE, National Institute for Health and Care Excellence; ONCOTYROL, Center for Personalized Cancer Medicine (Innsbruck, Austria); OECD, Organisation for Economic Co-operation and Development; PACE, Patient and Clinician Engagement group; PHARMGKB, Pharmacogenomics Knowledgebase; SBU, Swedish Agency for Health Technology Assessment and Assessment of Social Services; SMC, Scottish Medicines Consortium; SMDM, Society for Medical Decision Making; WHO, World Health Organization; ZINL, Zorginstituut Nederland.

PRISMA diagram of publications identified in literature search

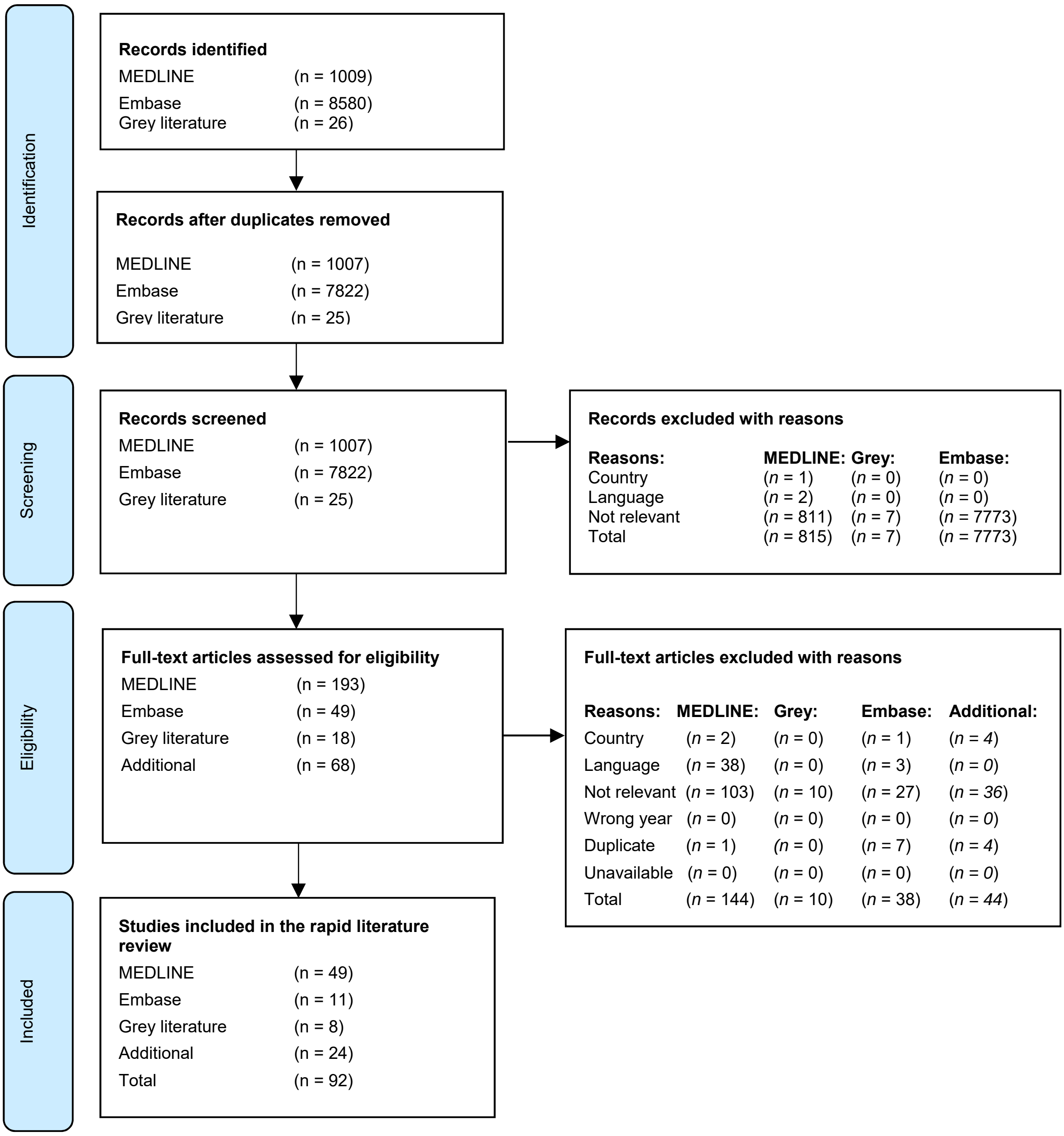

Supplement: Supplementary file 1 — Summary of literature review methods and PRISMA flow diagram. (PDF 138 kb) [file 12913_2017_2205_MOESM1_ESM.pdf]
