# Supplementary material for: Personalized medicine in Europe: not yet personal enough?
Source: BMC Health Serv Res. 2017 Apr 19;17:289. doi: 10.1186/s12913-017-2205-4 (PMC5395930; doi:10.1186/s12913-017-2205-4)
Supplement: Supplementary file 2 — Interview questionnaire. (PDF 130 kb) [file 12913_2017_2205_MOESM2_ESM.pdf]

Additional file 2 – interview guide

Questions asked varied depending on the stakeholder interviewed

DEFINITIONS

1. How would you define personalized medicine?
2. Does this apply to all kinds of therapeutic areas? *(prompt: oncology, multiple sclerosis, cardiovascular disease, diabetes)*
    a. If not, why?
    b. *(prompt for the different therapeutic areas)*

PATIENT SEGMENTATION

3. When talking about patient segmentation in a personalized medicine context, what does this refer to?
4. What factors would you consider for segmentation? (e.g. biomarkers, patient history, psycho-social factors, patient preferences/ behavior *(e.g. Lifestyle)*)
    a. Considering the factors you have identified, what are the potential benefits of each? (e.g. more effective and efficient treatment pathways, better information to sequence treatments, reduced harm to patients…)
5. Which factor (or factors) do you value the most and why? (e.g. biomarkers, hierarchy of factors, combination of factors, single factor)
    a. Would that be the optimal way for all therapeutic areas? (IF YES GO to Q52)
    b. If there is no optimal way, can you explain why?
6. What are the benefits and challenges of segmenting patients?

ASSESSMENT AND IMPLEMENTATION ***(Before starting this section ask whether interviewee is familiar with HTA, pricing, funding and reimbursement processes, if not provide definition/explanation)***

7. Are there specific HTA assessments or processes for personalized medicine in your country?
    a. Are these processes formal or informal?
    b. Who conducts them?
    c. How have they been validated?
    d. And what about patient segmentation?
8. Are there specific pricing and reimbursement guidelines with regards to personalized medicine that you know of and if so what are they
    a. Who is involved in these pricing and reimbursement decisions?

[If 53 & 54 are No, please skip to question 57]

9. What are the main criteria surrounding personalized medicine that are considered during the HTA, reimbursement, pricing and/ or funding assessments?
10. Do you think these criteria are appropriate for assessing personalized medicine? (
    a. If not appropriate, how should the criteria change?
11. Companion diagnostics provide information that is essential for the safe and effective use of a corresponding drug or biological product[1]. How would you value companion diagnostics? (*prompt: value of knowing, safety, effectiveness)*

---

[1] Definition from the FDA (US Food and Drug Administration), available at: http://www.fda.gov/MedicalDevices/ProductsandMedicalProcedures/InVitroDiagnostics/ucm407297.htm

12. Are there specific companion diagnostic technologies associated with personalized medicine that you know of?

If so, how are these companion diagnostic technologies assessed with regards to:

a. HTA?

b. Reimbursement?

c. Pricing?

i. Other (*prompt: regional or local assessments?*)

13. Are companion diagnostics assessed alongside personalized medicine or separately? *(Prompt: What are the implications of the current processes on patient access?)*

14. If a companion diagnostic gets a negative assessment (from an HTA or reimbursement process), can patients still have access to the drug in your country?

a. If so, how?

b. Are there any regional differences?

15. When thinking of your definition of personalized medicine (*repeat definition given*), have you come across a specific example that has been successfully implemented? If so what are the critical factors driving this success? *(prompt: approved and used in routine clinical practice)*?

Are there any specific unsuccessful experiences you would like to mention?
