# Supplementary material for: Personalized medicine in Europe: not yet personal enough?
Source: BMC Health Serv Res. 2017 Apr 19;17:289. doi: 10.1186/s12913-017-2205-4 (PMC5395930; doi:10.1186/s12913-017-2205-4)
Supplement: Supplementary file 3 — Definitions taken from the literature. (PDF 71 kb) [file 12913_2017_2205_MOESM3_ESM.pdf]

Additional file 3 – definitions taken from the literature

Author	Definition
Academy of Medical Sciences [1]	Stratified medicine is the grouping of patients based on risk of disease or response to therapy by using diagnostic tests or techniques.
APBI [7]	Stratified medicine: the tailoring of medical treatment to individual characteristics of each patient. Not literally the creation of drug or medical devices that are unique to a patient, rather the ability to classify individual into stratified subpopulations that differ in their susceptibility to (or severity of) a particular or their response to a specific treatment.
Becla [8]	Health care tailored to individual patient's need, based on the molecular analysis of gene expression, transcriptomics, proteomics, metabolomics, and analysis of the epigenome in individual patient, increasingly amplified with systems biology models.
Blair [9]	Stratified medicines are used with groups of patients with similar disease or response characteristics (…) [stratified medicine] is a separate concept to personalized medicine in which the characteristics of individual patients are considered.
Burock [10]	Requires the development of targeted agents either alone but most often in combination.
Burton [11]	Stratified medicine, the tailoring of intervention to a particular molecular understanding of the disease in an individual or their family.
Callaway [12]	This form of personalized medicine tailors treatments on the basis of molecular and genetic characteristics of a patient's cancer cells, potentially improving the treatment's outcome. (…) This form of stratified medicine uses genetic information to group patients according to their likely response to a particular treatment.
Cancer Research UK [13]	The ability to classify cancers according to their genetic make-up holds huge potential in delivering effective, personalized therapies that target specific pathways and mutations – stratified medicine.
Cho [14]	Personalized medicine is the ability to segment heterogeneous subsets of patients whose response to a therapeutic intervention within each subset is homogeneous.<br>US NIH: personalized medicine is an emerging practice of medicine that uses an individual's genetic profile to guide decisions made in regard to the prevention, diagnosis, and treatment of disease.<br>US FDA: personalized medicine as the best medical outcomes by choosing treatments that work well with a person's genomic profile or with certain characteristics in the person's blood proteins or cell surface proteins.<br>PCAST: Tailoring of medical treatment to the individual characteristics of each patient (…) personalized medicine is the ability to classify individuals into subpopulations that differ in their susceptibility to a particular disease or their response to a specific treatment.

Author	Definition
Ciardello [15]	The tailoring of medical treatment to the characteristics of an individual patient and moves beyond the current approach of stratifying patients into treatment groups based on phenotypic biomarkers.
Damato [16]	The tailoring of therapy to the needs, wishes, fears, and condition of the patient, also taking account of the individual's circumstances.
Denford 2014 [17]	The tailoring of medical treatment to the individual characteristics of each patient…to classify individuals into subpopulations that differ in their susceptibility to a particular disease or their response to a specific treatment.
Duburs [18]	The definition and understanding of any disease on molecular level for each individual or group of individuals (personalized diagnosis) ideally leading to the design of a drug that efficiently counteracts or prevents any molecular dysfunction, i.e. a personalized drug without side effects.
EC 2010a [19]	To provide the right diagnosis leading to prevention or treatment at the right dose to the right patient at the right time. Rather than having a unique treatment for each individual, patients can be sub-divided into groups based on their individual biological characteristics. By this stratification of patients with biomarkers, preventive and therapeutic interventions can be tailored to this particular group.
EC 2010b [20]	A medical model using molecular profiling technologies for tailoring the right therapeutic strategy for the right person at the right time, and to determine the predisposition to disease at the population level and to deliver timely and stratified prevention.
EC 2013 [21], EUAPM [2]	A medical model using molecular profiling for tailoring the right therapeutic strategy for the right person at the right time, and/or to determine the predisposition to disease and/or to deliver timely and stratified prevention.
Faulkner [22]	The use of genetic or other biomarker information to improve the safety, effectiveness, and health outcomes of patients via more efficiently targeted risk stratification, prevention, and tailored medication and treatment-management approaches.
Fiuzat [23]	The practice of obtaining non-obvious information, such as biomarkers, from an individual patient for the purpose of guiding therapeutic decisions tailored to that patient's needs.
Fricker [24]	Involves finding the driving mutations of individual patient's tumours and then matching them up to targeted therapies.
Garfield [25]	The tailoring of medical treatment to the individual characteristics of each patient. It does not literally mean the creation of drugs or medical devices that are unique to a patient but rather the ability to classify individuals into subpopulations that differ in their susceptibility to a particular disease or their response to a specific treatment. In the UK, personalized medicines are commonly, though not always, referred to as 'stratified medicines'.
Ginsburg [26]	Makes use of individual patient characteristics that are not routinely assessed in traditional medical practice today, such as genetic and genomic data as well as a patient's values and personal circumstances.

Author	Definition
Girard [27]	Targeted treatment tailored to the genetic makeup of individual tumours – a concept called personalized medicine.
Horgan [28]	A targeted approach to the prevention, diagnosis and treatment of disease based on an individual's specific profile.
Jakka [29]	Using genetic profiles to identify the best possible drug and therapy for a given patients and reducing adverse effects (goal of personalized medicine).
Jonsson [30]	Targeted cancer therapies are drugs or other substances that block the growth and spread of cancer by interfering with specific molecules involved in tumours growth and progression. Personalized medicine is a form of medicine that uses information about a person's genes, proteins, and environment to prevent, diagnose, and treat disease.
Lenz [31]	Personalised medicine refers to the tailoring of medical treatment to the individual characteristics of each patient; to classify individuals into sub-populations that differ in their susceptibility to a particular disease or their response to a specific treatment so that preventative or therapeutic interventions can then be concentrated on those who will benefit, sparing expense and side effects for those who will not.
Leopold [32]	Also known as co-dependent or stratified technologies, are defined as the treatment plan based on molecular screening and other test that suggest which regime will be most effective in specific patients. It is an approach to improve the use of medicines by using diagnostic testing (including genetic testing) to maximise clinical benefits and cost-effectiveness.
Louca [33]	Personalized medicine encompasses not only tailor-made drugs at the correct dose for the right patient, but also incorporates management of our personal data and clinical information. (Stratification of the patients' responses within groups might be more practical and manageable as a first step to personalized medicine before we are technologically and scientifically advanced to apply personalized medication. (…) Stratified medicine and the potential use of biomarkers are foreseen to have a major effect on both clinical practice and the development of new drugs and diagnostics).
Mathieu [34]	The use of a patient's biological, genetic and genomic information with a view to proposing the most effective and most appropriate treatment and/or dosage of medication.
Moch [35]	To understand the specific characteristics underlying a particular individual's disease and then match therapy to the molecular characteristics of this case. The right drug, the right dose, for the right patient, at the right time is the goal of personalized medicine.
Nature editorial [36]	Personalized medicine aims to use the latest genomic knowledge and technologies to tailor treatments to individuals.
Payne [37]	Personalized medicine is becoming a practical reality with the targeting of medicines by using a biomarker or genetic based diagnostic to identify the eligible patient population.

Author	Definition
Rogowski [38]	Interventions which seek to improve stratification and timing of health care by utilizing biological information and biomarkers on the level of molecular disease pathways, genetics, proteomics as well as metabolomics in addition to clinical patient characteristics.<br>Physiology based personalized medicine - detecting differences in the physiological characteristics of patients to target medical interventions.<br>Preference based personalized medicine - involves better information about a patients likelihood to respond to a treatment or to develop adverse effects, in addition to objective health benefit, such data adds to the information available to patients which could be used to better incorporate subjective preferences into treatment choices.
Schleidgen 2013a [3]	Identifying genetic, phenotypic, or environmental factors, which affect the subpopulation's health risks and help to find the most appropriate type and dose of medication and/or intervention. (…) [personalized medicine] understood as biomarker-based targeted treatment or prevention.
Schleidgen 2013b [39]	PM seeks to improve stratification and timing of health care by utilizing biological information and biomarkers on the level of molecular disease pathways, genetics, proteomics as well as metabolomics.
Tsimberidou [40]	Personalized or individualized medicine.
Verma [5]	Takes into account an individual's genetic make-up and disease history before developing a treatment regimen. (…) Personalized medicine is the right intervention for the right patient at the right time.
Zimmern [41]	The idea that medicines and other health technologies including the prediction of individual risk may be customised to each person's specific genetic, physiological or psychological characteristics.

ABPI, Association of the British Pharmaceutical Industry; EC, European Commission; EUAPM, European Alliance for Personalised Medicine; WHO, World Health Organisation
